# A Microbial Community Ecology Perspective on the Gut-Microbiome-Brain Axis

**DOI:** 10.3389/fendo.2020.00611

**Published:** 2020-09-02

**Authors:** Els van der Goot, Francjan J. van Spronsen, Joana Falcão Salles, Eddy A. van der Zee

**Affiliations:** ^1^Molecular Neurobiology, Groningen Institute for Evolutionary Sciences, University of Groningen, Groningen, Netherlands; ^2^Microbial Ecology Cluster, Groningen Institute for Evolutionary Sciences, University of Groningen, Groningen, Netherlands; ^3^Department of Pediatrics, Beatrix Children's Hospital, University Medical Center Groningen, Groningen, Netherlands

**Keywords:** microbiome, phenylketonuria, brain, behavior, probiotics

The gut microbiome is the collection of all microbial cells and associated genetic material present in the digestive tract of a host. Its composition depends on the gut environment and nutrition provided by the host (1). In turn, the microbes help the host by metabolizing complex nutrients, protecting against pathogens and priming their immune system (1, 2). In fact, the influence of the gut microbiome on host development goes beyond nutrition and immune response, by also regulating host behavioral and neurological responses (3, 4).

Microbiome-associated phenotypes can be seen as means for adaptation and natural selection, presenting an accessible point on which selection could work to tweak host-phenotype in case of changes in environmental conditions (5). From an animal perspective, the microbiome-associated phenotypes gained interest when Bercik et al. showed that fecal matter transplantation could direct strain-specific behavior of the recipient toward strain-specific behavior of the donor (6). Since then, a growing body of evidence has supported the effects of the microbiome on brain and behavior and the concept of a gut-microbiome-brain axis [see for reviews (3, 4)].

Although the microbiome-gut-brain-axis has been implicated in the pathophysiology of various (mental) diseases (3, 4), it is often an overlooked aspect in many (metabolic) disorders associated with behavioral deficits and treatments on a dietary basis. Which is remarkable, as diet is one of the main determinants of the gut microbiome and affects the (development) of cognitive (dys)function (7, 8). Moreover, beneficial effects of probiotic treatment on cognition have been reported for pathological conditions such as irritable bowel syndrome and coeliac disease (9).

Despite the growing interest in gut microbiome, the ecological aspects associated with these microbial communities are often not considered in the interpretation of the data, although they might contribute to the great variability in host performance often observed after microbiome manipulation, potentially leading to inconclusive interpretation of the data (10, 11). For instance, as communities, the gut microbiome is not static, being subjected to large fluctuations that reflect interactions among resident and transient microbial species and the host. From an ecological perspective, these interactions are driven by rules associated with microbial succession, as they reflect changes in community composition in response to processes such as selection and drift (12). Moreover, approaches targeting microbiome manipulation, including the development of probiotics (the “good” bacteria) or prebiotics (food for probiotics), require unraveling the ecological principles controlling microbial invasion, where mechanisms associated with microbial diversity and resource competition can help predicting the outcome of these strategies (11).

Despite the potential of using microbiome-related strategies to improve current treatment and neurological outcome of (metabolic) disorders, we argue that unraveling the ecological principles associated with the community dynamics is crucial, and subsequently a prerequisite to ensure the success in microbial-based treatments. To put our opinion in context, we will use the metabolic disorder phenylketonuria (PKU) to illustrate and explain how general concepts of community dynamics and resource availability, eco-evolutionary aspects and microbial invasions, apply in situations where the environment of the gastrointestinal tract is challenged and diet or probiotics are used to prevent neurological problems.

## The Gut Microbiome in the Context of PKU

PKU is an enzymatic deficiency of the hepatic phenylalanine hydroxylase which results in dramatically increased levels of Phe (>600 μmol/L). It can reach levels that are considered to be toxic for the brain, leading to severe intellectual disability (13–15). The most common treatment is to restrict the intake of natural protein in the diet, thus preventing high Phe levels in the brain, while supplementing with amino acids and essential micronutrients to avoid deficiencies (15–17). When followed early and continuously, this treatment is very effective in keeping Phe levels within an acceptable range (360–600 μmol/L), preventing severe high-Phe associated intellectual disability (15). Nevertheless, in many PKU patients with normal cognitive function Phe levels still influence brain performance (18). Alternatively, it has been shown that inconsistencies in neurocognitive, psychosocial and metabolic consequences of PKU remain, despite treatment (19–22).

It has been shown, that elevated Phe levels are not only present in the blood and brain, but are also manifesting in the gut (23). The microbes in the gut environment are constantly competing for resources, which become available either through host nutrition or the by-products generated by the microbial chemical food webs (24, 25). Together, the selective pressure exerted by the available resources and biotic interactions pressure play important roles in determining the microbiome composition in the gut of a given host (12, 26, 27). This means that the microbiome of PKU patients is constantly challenged by situations ranging between two extremes, depending on adherence to treatment; untreated with altered amino acid profiles and high Phe-levels, or low Phe-levels accompanied by a change in resource availability due to the strict dietary requirements. As the microbiome is adaptive in nature, this is likely to result in an altered microbial community. In both situations, alterations are likely to cause a less diverse microbiome, as Phe has been shown to be toxic to certain cell types (neurons), and resource restriction (natural protein) will challenge microbial species that either rely on these resources or are vulnerable to it, making it less likely to establish or to be successful (survival). Results from both PKU mice and patients show that, indeed, the PKU-associated microbiome is often less diverse and more variable between individuals, indicating dominance of a few species within a community (28–31). Moreover, studies that have examined prebiotic supplementation in PKU infant formula or the prebiotic properties of medical foods (glycomacropeptide) showed promising results in maintaining or increasing microbial diversity, indicating that altered resources might influence microbiome diversity in PKU (30, 32).

From a microbial ecology standpoint, the consequences of a less diverse microbiome include the community susceptibility to disturbances. Ecological theories predict that high diversity acts a biotic barrier, contributing to a stable microbiome or promoting microbiome resilience, capable of returning to the original, healthy state, upon disturbance. Thus, a reduction in gut microbial diversity significantly limits the ability of the microbiome to withstand major shifts, potentially leading to alternate, diseased, stable states (33, 34).

Another importance consequence of reduction in gut microbiome diversity or shifts in composition in PKU patients is the associated changes in the metabolite profiles of the microbiome, potentially modulating the chemical food web, thus influencing stability, as well as the molecules involved in microbiome-gut-brain signaling and brain functioning (35–38). In general, although many functions carried out by the microbiome show functional redundancy, i.e., that multiple populations are capable of carrying out that function, variability in the observed function after microbiome manipulation is greater than the change in gene frequency (10). This means that the interactions within the community strongly impact the functionality of the microbiome. As these functions include production of neurological signaling molecules, a dysfunctional microbiome could lead to behavioral symptoms. In case of dietary treated pathological conditions like PKU, the functionality of the microbiome could therefore impact the neurocognitive, psychosocial and metabolic outcome, despite a highly demanding diet. To optimize the outcome, and thus improving quality of life, a promising alternative would be the development of probiotics that could promote microbial diversity and microbiome functionality. Such approaches have been successfully used in other metabolic diseases (obesity related insulin insensitivity and type 2 diabetes), which are also associated with a dysbiotic gut microbiota and reduced microbial complexity (39, 40).

## Eco-Evolutionary Aspects Associated With Probiotic Supplementation

Probiotic supplementation for PKU can serve two purposes—it can be used to escape behavioral problems associated with treated PKU and it can be utilized to lower absorption of Phe from the gut by utilizing microbial metabolism. Although it has been shown that colonization is not necessary for probiotic action, interactions with the commensal microbiota will make supplementation less controllable and might lead to the unpredictable effects (41). Colonization of a desired probiotic or microbial consortia will therefore lead to longer lasting and more reproducible effects. Thus, for successful probiotic supplementation, general concepts such as community dynamics, microbial invasions and colonization are important to take into consideration to increase treatment effectiveness and safety.

**Figure 1:**
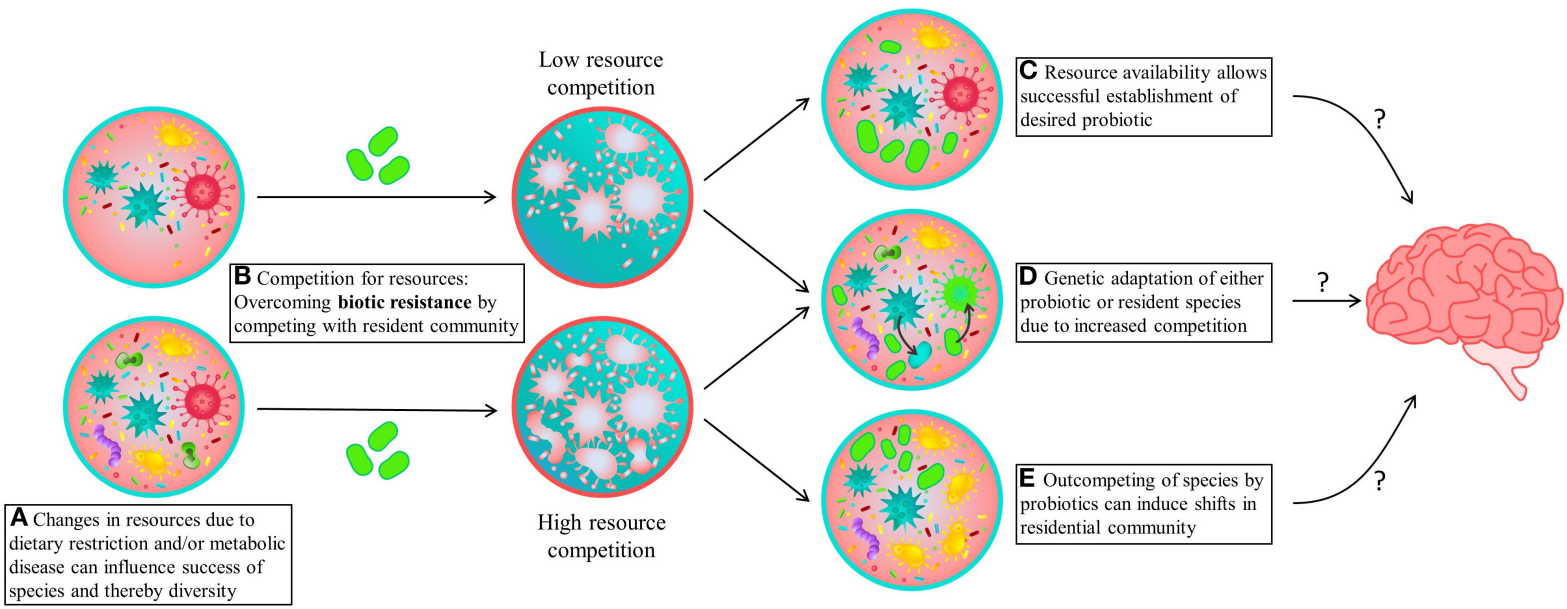
A schematic overview of potential consequences of probiotic supplementation. **(A)** An altered resource availability due to dietary restriction. **(B)** When presenting a probiotic (indicated by green microbes), these species will have to compete for available resources (represented by the blue color). **(C)** When the microbial diversity is low, resource availability will be sufficient for successful establishment and growth of the desired probiotic. However, when the microbial diversity is high, it usually correlates with high resource utilization and thus lower resource availability for the invading probiotic, leading to high competition and lack of establishment. This increased competition can either lead to **(D)** (genetic) adaptation of either the probiotic or resident species and **(E)** permanent or transient changes in the composition and functionality of the resident species, which in case of keystone species could lead to dramatic shifts in microbial community. These community dynamics could therefore lead to unpredictable changes in microbiome composition and potentially different effects on brain functioning then initially predicted from *in vitro* studies.

From an ecological perspective, probiotic supplementation can be studied in the context of microbial invasions, where the probiotic non-indigenous strain is introduced in large numbers into an existing community [(11, 42); Figure 1]. For microbial invasion to be successful, the invader has to overcome both abiotic (i.e., environmental factors like pH and temperature) and biotic resistance imposed by the resident community (Figure 1B). Ecological theory predicts that effectiveness and success of a probiotic treatment depends on the ability of the probiotic strain to invade and colonize the gut—which is correlated with their high growth rates, phenotypic plasticity and genetic diversity—but also depends on its capacity to compete for resources in the presence of the native gut microbiome (11, 43, 44). It has been shown that the success of invasion—in this case, the establishment of a probiotic strain—is negatively correlated with microbiome diversity (11, 45, 46). Specifically, diverse microbial communities explore the metabolic resources available in the gut in a more efficient manner, thus limiting the number of niches available for invaders (probiotic) to get established. On the other hand, when food resources are not fully consumed and invading species are capable of utilizing empty niches, the chances of establishment and growth are high (Figure 1C). In PKU, dietary restrictions and/or the influence of changes in amino acid profiles are likely responsible for lower species diversity due to altered resource availability, as opposed to liberalized dietary restrictions (Figure 1A). Thus, from the PKU perspective, the observed low gut microbiome diversity might increase the chances of probiotic establishment, although the altered resource availability intrinsic to PKU diet could prevent establishment and survival of the desired probiotics. Additional strategies that increase the probiotic's competitive ability, such as the use of prebiotics that stimulate the growth of the probiotic strain, or high phenotypic plasticity that ensure quick adaptation, might increase the chances of successful colonization therefore improving treatment effectiveness.

Examining microbiome dynamics during colonization (whether is it permanent or not) or whether the microbes were prone to adapt (mutate) within the gut-environment is important to determine safety of a given probiotic (47). In PKU, recent scientific advances give rise to the use of genetically modified probiotics to lower the absorption of Phe from the gut by relying on microbial metabolism (47–50). However, although they have shown to be stable as probiotics, little is known about how these supplements affect the ecological and evolutionary dynamics of commensal microbiota. Even with successful and beneficial introduction of the probiotics, the effects on the resident community, and subsequently behavior, might be more unpredictable—an aspect that is inherent to all microbial invasions (34, 51). Moreover, in situations in which the invader needs to compete for available resources, this increases the selective pressures and the propensity for horizontal gene transfer, which could lead to adaptation of either the probiotic or resident species (Figure 1D) (52–55). In the context of genetically modified bacteria, as for instance the Phe lowering probiotic, this could lead to integration of the modification in other (commensal) species, risking unfavorable expression of the given genes. Additionally, recent developments have shown that the probiotic properties of certain probiotic strains might be attributed to genes that induce mutational patterns that increase the risk of developing colorectal cancer (56). Thus, a systematic search for naturally occurring gut microbiome strains capable of degrading Phe might represent a more sustainable solution toward personalized medicine, where the evolutionary principles of the gut microbiome are considered (57).

Lastly, ecological principles associated with community dynamics might influence the outcome of microbiome manipulations due to the intricate relationship among microbial populations and their chemical food web (34, 51). Due to these community dynamics and its effect on resource availability, successful invasions can displace or shift resident taxa and alter community function, affecting multiple connections within the network (25, 58, 59). For instance, loss of a keystone species, one that is responsible for many connections in a chemical network, would make the community prone to collapse or create dramatic shifts in composition and function (Figure 1E). Microbiome alterations due to pre- or probiotics can therefore lead to changes in bacterial (metabolite) profile, resulting in different effects than predicted by *in vitro* studies, and to large impacts on the functionality of the entire microbiome. This could also explain why microbiota transfer therapy shows promising results, whereas supplementation of pre- and probiotics leads to variable results (60, 61). Unraveling the dynamics on composition, functionality and potential interactions within the gut microbiome are thus crucial to developing successful microbial based treatments and predicting its effects on microbiome functioning and cognitive outcome.

## Concluding Remarks

Microbial-based strategies for relieving neurological symptoms in various disorders are currently being studied more extensively. Nevertheless, additional research is needed to gain insight in the evolutionary and ecological microbial community dynamics. These dynamics play a critical role in the stability, composition and functional diversity of our gut microbiota, and thus the safety and success of probiotic treatment. Moreover, these ecological principles might explain the discrepancies found between animal and human studies, where results in the often more complex (human) microbiota are less profound and do not lead to the desired outcomes after microbiome manipulation (62, 63). Examining bacterial metabolite profiles resulting from the microbiome could uncover the exact mechanisms by which the gut microbiome influences the brain and hence many behavioral domains. It then could be used to develop personalized probiotic supplementation for disorders requiring dietary treatment, including PKU.

## Author Contributions

EG, JF, and EZ came up with the original concept and wrote the paper. EG had the primary responsibility for the final content. All authors read and approved the final manuscript as submitted and contributed in designing the final concept.

## Conflict of Interest

The authors declare that the research was conducted in the absence of any commercial or financial relationships that could be construed as a potential conflict of interest.
